# Why do patients take part in research? An overview of systematic reviews of psychosocial barriers and facilitators

**DOI:** 10.1186/s13063-020-4197-3

**Published:** 2020-03-12

**Authors:** Rebecca Sheridan, Jacqueline Martin-Kerry, Joanna Hudson, Adwoa Parker, Peter Bower, Peter Knapp

**Affiliations:** 1 University of York, York, UK; 2 Kings College London, London, UK; 3 University of Manchester, Manchester, UK; 4 University of York and the Hull York Medical School, York, UK

**Keywords:** Recruitment, Research participation, Psychosocial, Systematic review, Overview, Consent

## Abstract

**Background:**

Understanding why people take part in health research is critical to improve research efficiency and generalisability. The aim of this overview of systematic reviews was to identify psychosocial determinants of research participation and map them to psychological theory and empirical recruitment research, to identify effective strategies to increase research participation.

**Methods:**

Qualitative and quantitative systematic reviews were systematically identified. No date or language limits were applied. Two reviewers independently selected reviews. Methodological quality was rated using AMSTAR, and poor-quality reviews (scoring 0–3) were excluded. Barriers and facilitators were coded to psychological theory (Theoretical Domains Framework) and empirical recruitment research (recruitment interventions that had been subjected to randomised controlled trial evaluation).

**Results:**

We included 26 systematic reviews (429 unique primary studies), covering a wide range of patient populations and health settings. We identified five groups of facilitators, of which three were dominant (potential for personal benefit, altruism, trust) and appear to be relevant across research setting and design. We identified nine groups of barriers, which were more dependent on the particular study (context, population, design). Two determinants (participant information, social influences) were found to be both barriers and facilitators. Barriers and facilitators could be coded to the Motivation and Opportunity components of the Theoretical Domains Framework; only one was coded to a Capability component. There was some overlap between psychosocial determinants and empirical recruitment research, but some barriers and facilitators had not been tested at all.

**Conclusions:**

Identifying effective recruitment strategies could increase the efficiency and generalisability of primary research. We identified a number of barriers and facilitators that could be addressed by researchers. There is a need for more research to identify effective recruitment strategies that draw on the psychosocial facilitators and barriers identified in this overview.

## Background

Research is essential to the development of improved health care; however, the recruitment of participants remains low [1–3]. This is a particular problem for randomised trials, which test the effectiveness of interventions aimed at prevention, diagnosis, screening or treatment [4]. Suboptimal recruitment can result in underpowered and inconclusive studies, increased research costs and delays as well as unrepresentative sampling [1, 5]. There is a need to better understand the influences on participation in health research, particularly trials, and to identify areas and strategies for intervention. Accordingly, the PRioRiTy study, a James Lind Alliance Priority Setting Partnership, recently concluded that one of the most pressing recruitment questions was to determine what motivates participation [6].

Research participation is determined by psychosocial factors (that is, the interrelationship of social factors and individual cognitions and behaviours) acting as barriers or facilitators to impede or increase individuals’ willingness to take part. These determinants will vary conceptually, including instrumental (e.g. receiving financial compensation), attitudinal (e.g. being motivated by the potential for societal benefit), cognitive (e.g. believing that health care practitioners are virtuous) and emotional (e.g. fearing treatment change) components. Important social influences are likely to include the opinions of family and others whose views are valued. A number of systematic reviews have been undertaken to collate barriers and facilitators reported in primary studies, with most reviews focusing on specific conditions or patient groups.

The challenges experienced in recruitment have stimulated the production of a wide range of interventions to increase recruitment rates. Often these have been evaluated within SWATs (Studies Within A Trial), using trial methods to provide rigorous evidence of impact. Regarding the topic of recruitment [7, 8], for recruitment to trials, SWATs have been meta-analysed by Treweek et al. (2018) [8]; for recruitment to health research more generally, a systematic review was last undertaken in 2007 [7]. Notable in the Treweek review was that, despite a significant number of embedded trials (*n* = 68) and a range of intervention types (*n* = 72), in many cases there was no clear link between the tested intervention and reasons underpinning decisions to take part [8]. Therefore, there was an opportunity to review and collate a substantial evidence base on psychosocial determinants of research participation, and to look for features in the evidence that are generic or more context-specific. Making links between determinants and theory and recruitment interventions could strengthen the potency of interventions and, as a corollary, reduce levels of ‘research waste’ created by the evaluation of interventions without a clear rationale for possible effect.

The aims of this research therefore were to:

- Undertake an overview of systematic reviews of psychosocial determinants of research participation amongst patients and the public
- Summarise the reported determinants thematically and as barriers or facilitators
- Map these determinants to a behaviour change theoretical framework
- Map these determinants to interventions intended to increase participation in research.

## Methods

The review was reported in accordance with the Preferred Reporting Items for Systematic Reviews and Meta-Analyses (PRISMA) [9]. The review was registered in PROSPERO: http://www.crd.york.ac.uk/PROSPERO/display_record.php?ID=CRD42017062738.

### Data sources and searches

The search aimed to systematically identify reviews of psychosocial determinants of patient and public decisions on health research participation. The strategy was developed from one used in cancer trials [10] and was developed in MEDLINE (Ovid) before adaptation for other databases. No language, time or geographical limits were applied. Searches were limited to systematic reviews, using Database of Abstracts of Reviews of Effects (DARE) search strategies [11].

The following databases were searched 7th- 8th June 2016: MEDLINE, MEDLINE In-Process, Cumulative Index to Nursing and Allied Health Literature (CINAHL) Plus, Cochrane Database of Systematic Reviews (CDSR), Cochrane Methodology Register (CMR), DARE, Embase, Health Technology Assessment (HTA) database. PROSPERO was also searched for ongoing reviews. Results were imported into EndNote × 7 and de-duplicated. Reference lists of included articles were scanned, and forward citation searching was completed in Google Scholar. Searches were updated 4th December 2017 and 20th September 2019, retrieving a further 1197 and 1775 results, respectively (total 2972). (See Additional file 1 for the MEDLINE search strategy.)

### Inclusion and exclusion criteria

We included quantitative, qualitative or mixed methods systematic reviews reporting findings from studies exploring patient or public psychosocial determinants of health research participation. The focus of this review was on real research scenarios and not hypothetical research: work in this area often has mixed content, and so at least two thirds of primary studies within a review needed to involve actual research scenarios for inclusion. No language or publication status restrictions were applied. Systematic reviews were excluded if they only reported the characteristics of research participants, or if they were limited to health care practitioners’ views on the determinants of participation.

### Screening

Titles and abstracts were screened independently by two authors (RS and PK) using pre-defined criteria. All potentially relevant articles were retrieved and independently screened by RS and PK. Disagreements were resolved through discussion.

### Quality assessment

Two authors (RS and PK) used the assessment of multiple systematic reviews (AMSTAR) tool to assess the quality of reviews, and as an entry criterion [12]. Ratings were undertaken independently, and then an agreed score was reached through discussion. Items were scored 1 if the criterion was met and 0 if not met or unclear. One small modification to the recommended scoring was that, for criterion 5, articles only had to list included studies and not excluded studies (most reviews did not report excluded studies). A total AMSTAR score was calculated with review articles categorised as low (0–3), moderate (4–7) or high quality (8–11); low quality reviews (scoring 0–3) were excluded [13].

### Data extraction and analysis

Data extraction was undertaken using a pre-designed form. Extracted data included review aims, study design, participant details and key findings. Information was extracted by one reviewer (RS) and checked for accuracy by PK, except for key findings, which were independently extracted by both and reconciled by consensus. It was anticipated that the systematic reviews identified would include a variety of study designs, and thus a narrative reporting method was used. RS first identified psychosocial themes reported in included reviews and then grouped the data within these categories, in consultation with PK; themes were considered to facilitate participation or act as a barrier, or to do both. We adhered to behaviour change guidance by inductively coding barriers and facilitators to research participation (RS, PK, JH), which were then considered in relation to two theories of behaviour change: (1) the Theoretical Domains Framework (TDF) and COM-B model, described below [14–16], and (2) empirical research on interventions intended to increase rates of trial participation [8].

The TDF provides a comprehensive account of 14 domains which influence a person’s behaviour; it is used here because research participation is a behaviour. These 14 domains have been shown to cluster into three overarching constructs: capability, opportunity and motivation, which are defined in the behavioural science literature as the COM-B model [15, 17]. The capability construct recognises how psychological and physical capabilities influence behaviour. It includes the following TDF constructs: knowledge; skills; memory, attention and decision processes; and behavioural regulation. The opportunity construct outlines how the social and physical environment shapes behaviour. It includes the following TDF constructs: social influences; and environmental context and resources. The motivation construct considers conscious and unconscious cognitive processes that influence behaviour. It includes the following TDF domains: social or professional role and identity; beliefs about capabilities; optimism; beliefs about consequences; reinforcement; intentions; goals; and emotion.

In order to map barriers and facilitators to research participation against empirical interventions intended to increase recruitment research, we drew on a relevant Cochrane review [8]. The review included 68 trials, organised under six categories: trial design; trial conduct; consent process; modification to information; recruiter or recruitment site interventions; and incentives. The available evidence for the six categories varies considerably, and the lack of evidence for some means there is considerable uncertainty about effectiveness. Of note, whilst the included studies assessed 72 different recruitment strategies, only seven were assessed by more than one embedded study.

## Results

**Fig. 1:**
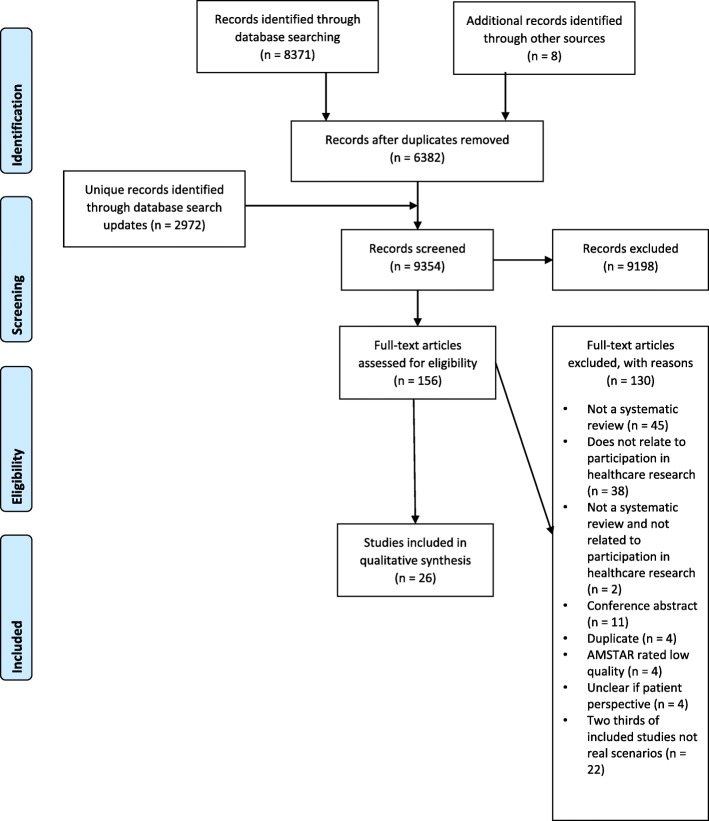
PRISMA flow diagram

We identified 6374 records and an additional eight through citation searching; 2972 further records were identified via the search updates, resulting in a total of 9354 articles. We retrieved 156 articles for full text review and finally included 26 articles. Exclusions are detailed in Fig. 1.

### Quality of the evidence

Six reviews (23.1%) were rated as high quality (AMSTAR score 8–11), and 20 reviews (72.9%) were of moderate quality (AMSTAR score 4–7) (see Table 1). Most involved a comprehensive literature search, employed duplicate study selection and data extraction and provided a list of included studies alongside their characteristics. The results of reviews were largely synthesised appropriately, and most authors stated whether there were any conflicts of interest. Just over half of the reviews assessed the scientific quality of the included studies, but only two assessed publication bias. The majority of reviews did not provide any evidence of a priori design, such as a published protocol.

**Table 1:** Characteristics of included systematic reviews

Author, year	Aim of review (as quoted)	Time frame of searches and date range of included studies	Population	Number of included studies (number in full review if different) Sample size Number of unique included studies	Included study design and data collection method	Subject of research participation	Location of included primary studies	AMSTAR score and category
Crane, 2017 [18]	The objective of this systematic review was to examine ethical issues surrounding research with **children and adolescents from their perspective as participants**	Time frame: dates not listed. Range: 2003–2014	Children and adolescents, majority with physical or mental illness	9 studies (23 in full review) *N* = 6326 Unique studies: 8 of 9	Qualitative: 4 Quantitative: 3 Mixed methods: 2 Methods: interviews, focus groups	Any phase vaccine trials	Sweden: 2 USA: 7	5, medium
Dhalla, 2013 [19]	The objective of this review article is to review **barriers to participation in actual preventative HIV vaccine trials**	Time frame: Cochrane Database for Systematic Reviews (no date), MEDLINE (1950–2012)/PubMed (no date), Embase (1980–2012), Google Scholar (no date). Range: 1994–2010. Range: 1995–2012	Adults 8 studies involving low-risk adults 12 studies involving ‘higher risk’ adults (e.g. intravenous drug users, gay men, sex workers)	20 studies *N* = 18,033 Unique studies: 8 of 20	Qualitative: not reported Quantitative: not reported Methods: focus groups, questionnaires, mixed methods, spontaneous reporting	Any phase HIV vaccine trials	Canada: 1 Kenya: 1 Spain: 1 Tanzania: 2 Thailand: 4 UK: 1 USA: 8 Multiple countries: 1 Not reported: 1	7, medium
Dhalla, 2014 [20]	The objective of this review article is to better understand **motivators to participation in actual preventive HIV vaccine trials** in terms of perceived social and personal benefits to such participation, as construed at these levels	Time frame: dates not listed. Range: 1997–2011	Adults. 9 studies involving low-risk adults 5 studies involving ‘higher risk’ adults (e.g. intravenous drug users, gay men, sex workers) 6 studies involving a mix of high- and low-risk adults 1 study unknown risk	21 studies *N* = 32,825 Unique studies: 11 of 21	Qualitative: not reported Quantitative: not reported Methods: questionnaires, interviews, telephone hotline and focus groups	Any phase HIV vaccine trials	Canada: 1 Italy: 1 Spain: 1 Tanzania: 2 Thailand: 6 UK: 1 USA: 6 Multiple countries: 3	6, medium
Fayter, 2007 [10]	Our aim was to undertake a systematic review of the relevant literature relating to the **barriers, modifiers, and benefits involved in participating in RCTs of cancer therapies** as perceived by health care providers and patients	Time frame: 1996–2004 Range: 1996–2004	Patients (adults and children) diagnosed with various cancers	37 studies (56 in full review) *N* = 25,788 (plus an unreported number from 4 studies) Unique studies: 23 of 37	Qualitative: not reported Quantitative: not reported. Methods: surveys, focus groups, chart review, case controlled studies	Randomised controlled trials (excluding solely phase I or II)	Australia: 3 Canada: 1 Denmark: 1 Finland: 1 Italy: 1 Netherlands: 1 Sweden: 1 UK: 12 USA: 15 Multiple countries: 1	8, high
Fisher, 2011 [21]	This review takes a different focus and considers the **reasons that parents accept or decline an invitation to enrol children of any age in clinical research**	Time frame: Scopus 1960 to Feb 2010; Web of Knowledge 1971 to Feb 2010 Range: 2001–2011	Parents/caregivers of children invited to take part in research Two thirds of studies involved children with life-limiting or life-threatening conditions including cancer and diabetes	16 studies *N* = 365 (plus an unreported number from one study) Unique studies: 10 of 16	Qualitative: 16 Quantitative: 0 Methods: interviews, focus groups, ethnography, content analysis of websites	14 trials, 2 unclear	Canada: 1 Gambia: 1 Malawi: 1 UK: 4 USA: 9	7, medium
Forcina, 2018 [22]	We aimed to conduct a systematic review of studies limited to AYA patients which assessed **attitudes and beliefs that influence cancer CT enrolment** to prioritize areas for future study and intervention	Time frame: inception to May 2017 Range: 2009–2016	Adolescent and young adult cancer patients aged 15–39 years	6 studies *N* = 754 Unique studies: 4 of 6	Qualitative: not reported Quantitative: not reported Methods: semi-structured interviews, questionnaires/surveys	Cancer clinical trials	USA: 1 Not reported: 5	6, medium
Gad, 2018 [23]	We conducted a literature review to determine (1) **the factors that influence*[cancer]*patients’ decisions to enter a phase I trial,** (2) patients’ perceptions of the information they receive when they are invited to participate in a phase I trial and (3) relatives’ perceptions of the information given to patients	Time frame: inception to April 2017. Range: 1995–2014	Adult patients diagnosed with various cancers	15 studies (37 in full review) *N* = 1313 Unique studies: 10 of 15	Qualitative: 4 Quantitative: 11 Methods: interviews, questionnaires, focus groups	Phase I trials	Canada: 1 Italy: 1 Japan: 2 UK: 3 USA: 8	10, high
Glover, 2015 [24]	To our knowledge there have been no reviews that specifically focus on **barriers or facilitators for Indigenous peoples’ participation in RCTs**. This paper aims to address that knowledge gap by presenting the findings of a systematic review of the literature on challenges and facilitators of participation in health RCTs amongst Indigenous people from New Zealand, Australia, Canada and the USA	Time frame: inception to March 2012. Date range: 1994–2011	5 studies with Indigenous or Aboriginal Australians, 4 studies with First Nation participants, 6 studies with Maori participants, 31 studies with Native Americans or Alaskan Natives The majority (*N* = 11) focused on cancer, 7 on diabetes, 6 on substance abuse and 22 on other conditions/factors	46 studies *N* = not reported Unique studies: 45 of 46	Qualitative: not reported Quantitative: not reported Methods: not reported	Randomised controlled trials	Australia: 5 Canada: 4 New Zealand: 6 USA: 31	5, medium
Grand, 2012 [25]	This review examines the relationship between the obstacles to **participation in cancer clinical trials** and accrual, **focusing wherever possible on clinical trials in radiation oncology**	Time frame: 1984 to 2009 Range: 1983–2007	Patients diagnosed with various cancers	20 studies (31 in full review) *N* = 13,681 Unique studies: 15 of 20	Qualitative: not reported Quantitative: not reported Methods: questionnaires, interviews, surveys, review of patient/trial records, focus groups	Oncology clinical trials	Not reported	5, medium
Gregersen, 2019 [26]	To systematically review and thematically synthesise the **experiences of patients and relatives when they have to decide whether or not to participate in a clinical oncology trial** and to provide knowledge about the decision-making process	Time frame: dates not listed Range: 2000–2016	Adult patients with advanced cancer	11 studies *N* = 203 Unique studies: 4 of 11	Qualitative: 11 Quantitative: 0 Methods: interviews, focus groups	Clinical trials	USA: 6 UK: 3 Japan: 1 Sweden: 1	5, medium
Hughes-Morley, 2015 [27]	Our aims in undertaking this review were firstly to systematically identify relevant qualitative studies describing **factors affecting recruitment of participants into depression trials**; and secondly to perform a meta-synthesis to identify common themes that describe factors affecting recruitment into depression trials, to develop a conceptual framework of factors influencing the decision to participate in depression trials	Time frame: ASSIA 1987 to April 2013; CINAHL 1937 to April 2013; Embase 1974 to April 2013; MEDLINE 1946 to March 2013; PsychInfo 1806 to April 2013 Range: 2007–2012	Patients with depression	4 studies (15 in full review) *N* = 1034 Unique studies: 4 of 4	Qualitative: 4 Quantitative: 0 Methods: questionnaire, interviews, focus groups	Randomised controlled trials	UK: 3 Multiple countries: 1	9, high
Liljas, 2017 [28]	This systematic review aimed to identify facilitators, barriers and strategies for **engaging ‘hard to reach’ older people in research on health promotion**; the oldest old (≥ 80 years), older people from black and minority ethnic groups (BME) and older people living in deprived areas	Time frame: 1990 to 2014 Range: 1996–2014	18 studies of BME older people (aged over 50 years), 3 studies with oldest old patients (80 years and over) and 2 studies of older people in deprived areas	23 studies *N* = not reported Unique studies: 23 of 23	Qualitative: 12 Quantitative: 10 Mixed methods: 1 Methods: surveys, questionnaires, interviews, focus groups	Not reported. Studies focused on health promotion	Canada: 1 New Zealand: 1 UK: 4 USA: 17	7, medium
Limkakeng, 2013a [29]	We carried out a systematic review of literature published between 1985 and 2009 to understand **Chinese patients’ motivations and concerns to participate in clinical trials**	Time frame: 1985–2009 Range: 2004–2008	Chinese adults between 18 years and 85 years 1 study relating to HIV vaccine trials and 1 relating to cancer, 3 studies non-specific	5 studies *N* = 645 Unique studies: 5 of 5	Qualitative: 3 Quantitative: 2 Methods: surveys, interviews	Clinical trials	USA: 3 China: 1 Singapore: 1	8, high
Limkakeng, 2013b [30]	The objective of this study was to conduct a systematic review and metasummary to evaluate what values, attitudes or beliefs on the part of **potential or actual research participants with emergent medical conditions influence participation in research**	Time frame: inception to 2011 Range: 2000–2009	Adult patients aged over 18 years 5 studies focused on suspected myocardial infarction patients, 3 on stroke patients, 1 on sudden cardiac near-death survivors and 5 on other emergency patients	14 studies *N* = 4003 (plus 1 study unclear) Unique studies: 12 of 14	Qualitative: 6 Quantitative (survey): 8 Mixed methods: 3 Methods: surveys, interviews	Not reported.	Primarily conducted in American and Western European contexts Number not reported	7, medium
Martinsen, 2016 [31]	The aim of the current report was to perform a systematic review of the current literature on participation motives, response rates and recruitment strategies in **research bronchoscopy studies with an emphasis on studies including COPD patients**	Time frame: dates not listed Range: 1998–2013	Patients with HIV, bronchoscopy patients, smokers, children with or without cystic fibrosis and parents	6 studies (7 in full review) *N* = 455 Unique studies: 6 of 6	Qualitative: not reported Quantitative: not reported Methods: interviews and questionnaires, focus groups, surveys	Not reported	The Netherlands: 1 UK: 3 USA: 1 Multiple countries: 1	5, medium
McCann, 2007 [32]	The aim of this review was to draw together qualitative and quantitative studies reporting **patients’ experiences of trial recruitment and participation** to provide a broad-based overview of the literature	Time frame: 1996–2005 Range: 1982–2005	Demographic data largely not reported. Range of trials including HIV, cancer, neonatal and myocardial infarction	32 studies *N* = 6068 Unique studies: 21 of 32	Qualitative: 12 Quantitative: 12 Mixed methods: 3 Systematic reviews: 5 Methods: interviews, questionnaires	Phase III trials	Denmark: 2 Europe: 1 Israel: 1 UK: 13 USA: 9 The Netherlands: 1 Multiple countries: 5	4, medium
McCann, 2013 [33]	Update of 2007 review—no new objective stated	Time frame: September 2005 to December 2010 Range: 2006–2010	Patients with a variety of conditions including cancer, epilepsy, stroke and pre-term labour. One paper discussed interviews with parents of children with leukaemia and 2 studies involved pregnant women or parents	11 studies (12 in full review) *N* = 290 Unique studies: 9 of 11	Qualitative: not reported Quantitative: not reported Methods: interviews, focus groups, observations	Randomised controlled trials	Australia: 1 Denmark: 1 UK: 7 USA: 1 Multiple countries: 1	7, medium
Nalubega, 2015 [34]	This review aimed to synthesize and present the best available evidence in relation to **HIV research participation in sub-Saharan Africa**, based on the views and experiences of research participants	Time frame: inception to July 2013. Updated in September 2014 Range: 2004–2014	All current or former adult HIV research participants from sub-Saharan African countries. 16 studies only involved women	21 studies *N* = not reported Unique studies: 18 of 21	Qualitative: 21 Quantitative: 0 Methods: focus groups, interviews, participant observation	Not reported	Kenya: 1 Malawi: 1 South Africa: 12 Tanzania: 4 Zimbabwe: 1 Multiple countries: 2	9, high
Nielsen, 2019 [35]	The aim of this study was to examine cancer patients’ perceptions of factors that may influence their **decisions on participation in phase I–III clinical drug trials**	Time frame: 2010–2016 Range: 2010–2013	Adult cancer patients	9 studies *N* = 236 Unique studies: 3 of 9	Qualitative: 9 Quantitative: 0 Methods: interviews, questionnaires	Cancer clinical drug trials	USA: 7 Japan: 1 Sweden: 1	5, medium
Nievaard, 2004 [36]	To assess the factors that may **influence a patient’s consent to participate in a clinical trial**	Time frame: 1980 to April 2002 Range: 1984–2002	Adult patients. 14 with cancer patients, 5 with HIV patients, 6 from other patient groups and 5 did not report the patient group	30 studies *N* = not reported Unique studies: 19 of 30	Qualitative: not reported Quantitative: not reported Methods: Not reported	6 randomised controlled trials, others not reported	Australia: 3 USA: 14 Western Europe: 13	5, medium
Nobile, 2013 [37]	The aim of this article is to review the literature addressing **actual and apparently healthy participants’ reasons to enrol in biobank studies** in order to see if some motives are unduly influencing the decision to participate	Time frame: inception to Jan to Feb 2012 Range: 2006–2012	Healthy adult participants. 4 studies involved just women	13 studies *N* = 1762 Unique studies: 12 of 13	Qualitative: 9 Quantitative: 4 Methods: interviews, focus groups and surveys	Not reported.	Australia: 2 Europe: 1 UK: 3 USA: 7	5, medium
Prescott, 1999 [38]	To assemble and classify a comprehensive bibliography of **factors limiting the quality,number and progress of RCTs**. To collate and report the findings, identifying areas where firm conclusions can be drawn, and identifying areas where further research is required	Time frame: 1986 to March 1996 Range: 1986–1996	Majority of studies involved cancer patients (*N* = 9), 2 studies concerning child health involved parents/caregivers	22 studies (27 studies in chapter) *N* = 15,295 Unique studies: 19 of 22	Qualitative: not reported Quantitative: not reported Surveys, trial data, questionnaires, structured interviews	Clinical trials, not phase I or phase II	Australia: 1 Canada: 1 France: 3 The Netherlands: 1 UK: 5 USA: 11	8, high
Quay, 2017 [39]	The aim was to identify barriers and facilitators to **recruitment of South Asians to health research studies** and associated strategies to improve participation	Time frame: January 2004 to April 2016 Range: 2004–2016	South Asian patients. Majority of studies involved patients with a condition, e.g. asthma or diabetes	10 studies (15 in full review) *N* = 3139 Unique studies: 8 of 10	Qualitative: 9 Quantitative: 6 Methods: surveys, interviews, focus groups, literature reviews	10 randomised controlled trials	Australia: 1 India: 1 UK: 7 USA: 1	8, high
Tromp, 2016 [40]	This systematic review attempts to answer the following research question: **What are motivating and discouraging factors for children and their parents to decide to participate in clinical drug research**?	Time frame: inception to March 2013. Updated August 2014. Range: 1997–2013	26 studies involved parents or caregivers/guardians, 5 involved children and 11 involved both. Included children aged between 6 and 21 Diverse research population but many involved oncology patients (11 studies) 39 studies involved people who had consented, 24 involved people who had dissented. 29 studies involved treatments with prospect of direct benefit	42 studies *N* = 5500 Unique studies: 33 of 42	Qualitative: 16 Quantitative: 26 Methods: questionnaires, registry analysis, focus group, interviews	Not reported	Not reported	7, medium
Van der Zande, 2018 [41]	The objective of our paper was to identify and systematically review all articles regarding **pregnant women’s reasons to participate in clinical research**	Time frame: dates not listed Range: 2013–2016	Pregnant/previously pregnant women	30 studies *N* = 7905, plus an unreported number from 1 study Unique studies: 28 of 30	Quantitative: not reported Qualitative: not reported Methods: interviews, focus groups, questionnaires, surveys, analysis of records	Observational studies and randomised controlled trials	UK: 10 USA: 7 Canada: 5 Australia: 2 China: 1 Ghana: 1 Ireland: 1 Italy: 1 Netherlands: 1 Pakistan: 1	5, medium
Woodall, 2010 [42]	We aimed to review the current literature on the nature of **barriers to participation across different mental health studies with a focus on whether there are specific gender-, age- and ethnicity-related barriers**	Time frame: 1990 to 2008 Range 1992–2008	Adult participants. 5 schizophrenia studies, 5 depression studies, 6 dementia studies and 5 where the illness was not specified	16 studies (49 in full review) *N* = 2033, plus an unreported number from 9 studies Unique studies: 15 of 16	Qualitative: not reported Quantitative: not reported Methods: surveys, interviews, recruitment	Not reported.	Australia: 1 Canada: 1 Germany: 1 Mexico: 1 Switzerland: 1 UK: 1 USA: 10	6, medium

### Characteristics of included studies

The 26 reviews incorporated a total of 489 relevant primary studies, of which 179 (36.6%) had been undertaken in the USA; 80 (16.4%) in the UK; 19 (3.9%) in Australia; 17 (3.5%) in Canada; 12 (2.4%) in South Africa; 10 (2.0%) in Thailand; whilst 28 (5.7%) had been undertaken in more than one country (see Table 1). Country of origin was not reported in the source review for 82 (16.8%) studies, and the remaining 62 studies had been undertaken in one of 23 countries. Of the 489 primary studies, 56 (11.5%) were included in more than one review, leaving a total of 429 unique studies.This degree of overlap in the primary studies is low, incorporating a covered area of 4.4% and a corrected covered area of 0.5% [43]. Six (23.1%) reviews [10, 21, 26, 27, 34, 35] explicitly stated that they included only qualitative studies; the remainder included both quantitative and qualitative research. The focus of reviews varied in terms of health setting and types of research participation. Sixteen (61.5%) reviews were limited to studies of trial participation [10, 18–20, 22–27, 29, 32, 33, 35, 38, 39], and the remaining ten either included a mix of primary research designs or the design was unclear [21, 28, 30, 31, 34, 36, 37, 40–42]. Fifteen (57.7%) reviews were related to specific health conditions or settings: cancer (*n* = 6), HIV (*n* = 3), mental health (*n* = 2), chronic obstructive pulmonary disease (COPD), emergency medicine, pregnancy and bio-banking (each *n* = 1). Four studies focused on child or adolescent participants and their parents/caregivers [18, 21, 22, 40]; one study focused on ‘hard to reach’ older patients [19]; and four reviews focused on ethnic minority groups [24, 28, 29, 39]. Fifteen reviews (57.7%) only included real research scenarios [19–21, 23, 24, 26, 28–30, 32–37]; whereas 11 (42.3%) included both real and hypothetical scenarios. Most reviews (19; 73.1% considered both facilitators and barriers to research participation; three (11.5%) were limited to facilitators and four (15.4%) to barriers. The reviews were published during 1999–2019; their included primary studies were published during 1982–2016. Characteristics are further detailed in Table 1.

### Identified psychosocial themes

#### Facilitators of research participation

A number of themes were identified which reported facilitators of research participation (see Table 2). The most commonly reported was perceived personal benefits, including the perception of therapeutic benefits, closer monitoring and access to new treatments [10, 20–22, 25–27, 29–37, 39–41].

**Table 2:** Identified psychosocial facilitators and barriers to research participation, mapped to the Theoretical Domains Framework (TDF) and tested recruitment interventions

Identified theme	Systematic reviews reporting the theme	Domain (components) of the TDF (from Cane et al., 2012) [14]	Interventions which probably affect recruitment to research (from Treweek et al., 2018) [8]	Interventions shown not to affect recruitment to research, or with uncertain effects (from Treweek et al., 2018) [8]
**Facilitators**				
Personal benefit (including therapeutic benefits; closer monitoring; access to new treatments; gaining knowledge of own health)	Reported in 20 SRs: Dhalla, 2014; Fayter, 2007; Fisher, 2011; Forcina, 2018; Grand, 2012; Gregersen, 2019; Hughes-Morley, 2015; Liljas, 2017; Limkakeng, 2013a; Limkakeng, 2013b; McCann, 2007; McCann, 2013; Martinsen, 2016; Nalubega, 2015; Nielsen, 2019; Nievaard, 2004; Nobile, 2013; Quay, 2017; Tromp, 2016; van der Zande, 2018	Optimism (Reflective Motivation)	Mentioning scarcity of trial places Positive framing of potential treatment benefits	Patient preference trial design
Altruism (including benefits to science; helping others)	Reported in 18 SRs: Dhalla, 2014; Fayter 2007; Fisher 2011; Forcina, 2018; Gregersen, 2019; Hughes-Morley 2015; Limkakeng, 2013a; Limkakeng, 2013b; Martinsen, 2016; McCann, 2007; McCann, 2013; Nalubega, 2015; Nobile, 2013; Nielsen, 2019; Nievaard, 2004; Quay, 2017; Tromp, 2016; van der Zande, 2018	Beliefs about consequences (Reflective Motivation)		
Confidence or trust in the physician or the research	Reported in 13 SRs: Crane, 2017; Grand, 2012; Gregersen, 2019; Hughes-Morley, 2015; Liljas, 2017; Limkakeng, 2013a; Limkakeng, 2013b; Martinsen, 2016; McCann, 2007; McCann, 2013; Nielsen, 2019; Nievaard, 2004; Nobile, 2013	Reinforcement (Automatic Motivation)	Endorsements of previous participants	
Low burden or convenient research	Reported in 4 SRs: Limkakeng, 2013a; Nobile, 2013; Tromp, 2016; van der Zande, 2018	Belief about consequences (Reflective Motivation) Social or Professional Role & Identity (Reflective or Automatic Motivation)	Opt-out consent method	Two-stage randomisation method (may increase perceived inconvenience to the participant)
Financial benefit or incentives	Reported in 3 SRs: Limkakeng, 2013a; Nalubega, 2015; Tromp, 2016	Goals (Reflective Motivation)	Financial incentives	
**Barriers**				
Fear and perceived risk (to health, of experimental treatment or adverse effects; to personal consequences)	Reported in 14 SRs: Dhalla 2013; Forcina, 2018; Fisher 2011; Grand, 2012; Hughes-Morley, 2015; Martinsen, 2016; McCann, 2013; Nalubega, 2015; Nielsen, 2019; Nievaard, 2004; Quay, 2017; Tromp, 2016; van der Zande, 2018; Woodall, 2010	Belief about consequences (Reflective Motivation)	Emphasising pain in information (−)	Emphasising risk in information
Practical difficulties (including additional procedures or appointments; transport; costs; work or caring responsibilities)	Reported in 13 SRs: Fayter, 2007; Forcina, 2018; Glover, 2015; Grand, 2012; Hughes-Morley, 2015; Liljas, 2017; Martinsen, 2016; McCann, 2007; Prescott, 1999; Quay, 2017; Tromp, 2016; van Der Zande, 2018; Woodall, 2010		Financial incentives Internet-based data collection (−)	Two-stage randomisation method (may increase practical demand) Email (not postal) invitations
Distrust of research or researchers (particularly amongst ethnic minorities)	Reported in 10 SRs: Glover, 2015; Hughes-Morley, 2015; Limkakeng, 2013a; Limkakeng, 2013b; McCann, 2007; Nalubega, 2015; Quay, 2017; Tromp, 2016; van der Zande, 2018; Woodall, 2010			
Aversion to randomisation	Reported in 7 SRs: Forcina, 2018; Hughes-Morley, 2015; McCann, 2007; McCann, 2013; Nievaard, 2004; Tromp, 2016; van der Zande, 2018	Environmental context and resources (Physical Opportunity)	Open trial design	Cluster trial design
Treatment preferences (for specific therapy; against placebo)	Reported in 5 SRs: Fayter, 2007; Grand, 2012; McCann, 2007; Prescott, 1999; Tromp, 2016	Reinforcement (Automatic Motivation)	Open trial design	Patient preference trial design
Stigma associated with health condition	Reported in 5 SRs: Dhalla, 2013; Hughes-Morley, 2015; Nalubega, 2015; Woodall, 2010; Quay, 2017	Social influences (Social Opportunity)		
Uncertainty (particularly in relation to trials; its links to randomisation)	Reported in 4 SRs: Fayter, 2007; Fisher 2011; Nievaard, 2004; Prescott, 1999	Belief about consequences (Reflective Motivation)		Patient preference trial design
Personal health	Reported in 4 SRs: Hughes-Morley, 2015; Liljas, 2017; Limkakeng, 2013b; Woodall, 2010	Emotion (Automatic Motivation)		
Desire for choice	Reported in 3 SRs: Grand 2012; Fisher 2011; Tromp 2016	Goals (Reflective Motivation)		Patient preference trial design
**Factors reported as facilitators and barriers**		Belief about consequences (Reflective Motivation)		
Influence of physician, family or friends	Reported in 11 SRs: Fayter 2007; Forcina, 2018; Hughes-Morley, 2015; Gad 2018; Gregersen, 2019; Liljas, 2017; Limkakeng, 2013a; Nielsen, 2019; Prescott 1999; Tromp, 2016; van der Zande, 2018	Belief about consequences (Reflective Motivation)	Endorsements of previous participants	
Information quality and participant’s knowledge of the research	Reported in 5 SRs: Crane, 2017; Fayter 2007; Forcina, 2018; Glover, 2015; Gregerson, 2019	Social influences (Social Opportunity)	Enclosing questionnaire on study method	Researcher reading out information (?) Easy-to-read consent form Optimising information through user testing or user feedback Brief patient information leaflet Providing information by phone Providing information by video (?) Providing audio record of recruitment discussion (?) Providing booklet on trial methods (?) Total or discretionary information disclosure (?) Educational package on study

Key: (−) negative effect on recruitment, (?) uncertain effect on recruitment

Whilst altruism was the second most commonly reported factor, discussed in terms of benefitting science [10, 20, 29–31, 34, 36, 37, 39–41], helping others [10, 20, 21, 26, 27, 30, 31, 34, 35, 37, 40, 41] or altruism more generally [22], this was sometimes linked to personal benefit [27, 31, 33]. For example, patients with depression were less likely to participate if it might risk their own mental health, despite wanting to help others [27]. Further, two reviews highlighted that the desire to help others was not always concerned with helping all people, but specifically benefitting people who were personally important [32, 37]. Finally, a review involving research with children and adolescents concluded that the importance of altruism depended on the child’s health state; altruistic motives were given as a primary reason for participation by parents with healthy children, but for parents whose children had life-threatening conditions, altruism was secondary [21].

The influence of others was also important. Potential participants’ confidence in the physician and/or the research was motivating [18, 23, 25–28, 30–33, 35–37]. Having a positive, trusting relationship with the doctor was commonly cited as a facilitator; for example, the idea that the ‘doctor knows best’ was expressed [38]. The opinions of family and friends also facilitated participation [27–29, 35, 40, 41].

The impact of the potential participant’s knowledge of trials and the quality of the study information was mixed. For example, knowing you could leave the trial increased participation [10], but one review highlighted that enhanced knowledge and understanding could decrease participation [32]. A study with children and parents highlighted the need for age-appropriate information [18], whilst another highlighted the need for cultural appropriateness [24]. However, knowledge could act as a barrier when too much complex information was provided [10, 22, 30] or when information was vague [36]. Gaining knowledge of their health condition was a participation facilitator for children [40] and those invited to biobank studies [37].

Financial benefits were discussed in three reviews, but did not appear to be a primary determinant [29, 34, 40]; rather, financial benefits were seen as an added bonus [34]. However financial constraints and costs could inhibit participation [28, 39, 40].

#### Barriers to research participation

Fear was identified as a barrier in a large number of reviews, often related to perceived risks of treatments or interventions being tested and possible side effects [19, 21, 22, 25, 27, 31, 33–36, 39–42]. Assessment of risk varied with the severity of the patient’s illness [21]; for example, patients with a life-limiting diagnosis were more tolerant of research risk, potentially because of the access that participation granted them to new medication [21]. This was also linked to a perceived lack of choice imposed by the terminal diagnoses: patients stated the view that there seemed no option but to participate [21, 26, 35, 40]. More specific fears regarding the safety of interventions were common in reviews of HIV vaccine trials [19, 29]: potential trial participants were concerned about vaccine efficacy, or whether it could increase their susceptibility to HIV [29]. Other fears included discovering their HIV status [34] or being reported to immigration [39].

Distrust in research was common across patient groups [24, 27, 29, 30, 32, 34, 39–42], but was particularly prominent amongst minority ethnic groups [27, 39], minority indigenous populations [24] and people in sub-Saharan Africa [34]. In one review, distrust was linked to a lack of knowledge and understanding [29]. Specific distrust concerns included potential breaching of privacy or confidentiality [24, 29, 42], being a ‘guinea pig’ [30, 40] and a general mistrust of researchers’ intentions [34]. Nevertheless, trust in the safety of research was also reported as a motivating factor [21, 40].

Treatment preference, either for or against a specific treatment, was a reported barrier in several reviews [10, 25, 32, 38, 40]. Preferences included not wanting to change medication or not wanting to receive a placebo or experimental treatment [38]. However, preference for a specific treatment could also be a facilitator; in one mental health systematic review participants wanted access to the non-pharmaceutical, talking therapies on offer [27].

Perceived stigma was a commonly reported barrier to recruitment to trials in HIV [19, 34] or mental health [27, 42]. People did not want others to know their HIV status or to assume it as a result of trial participation [19, 34]. In mental health studies, stigma was largely due to people not wanting to be perceived as ‘crazy’, ‘weak’ or ‘vulnerable’ [27].

Practical difficulties were highlighted including the perceived inconvenience of trial participation (for example, additional procedures and appointments) [19, 22, 27, 32, 38, 40], a lack of time [19, 28, 32, 38, 41], travel or transport issues [10, 19, 24, 25, 28, 31, 32, 38, 39, 41, 42], costs [10, 25, 28, 38–40], as well as employment [10, 39] or childcare responsibilities [10, 28].

Concerns about trial methods were highlighted as barriers, including the inherent uncertainty [10, 21, 36, 38] and randomisation [22, 27, 32, 33, 36, 40, 41]. Potential participants also stated concerns about possible unknown side effects [10] and uncertain treatment effectiveness [21, 38]. There was some evidence of confusion about the meaning of randomisation [32], whilst other reviews noted that patients understood the concept but felt that randomisation signified a loss of control [25, 38] or that the doctor should choose treatments based on clinical expertise. In contrast to the inhibiting effects of concern about trial methods and the practical implications of research, the perception of a trial as low burden or convenient tended to facilitate participation [29, 37, 40].

Whilst knowledge could facilitate research participation, a lack of knowledge and understanding of clinical research could have a negative effect [24, 29, 30, 40], and participants identified a need for more information [38]. This lack of knowledge was sometimes linked to limitations of the informed consent process [30].

Finally, the patient’s health state at the time of invitation to participate was important in some reviews. Some patients felt too ill to participate [27, 28, 42]; others who were happy with their current health were less likely to participate for fear of disrupting this [27]. However, adverse health could favour research participation. One review of trials in acute conditions found that patients in pain said they were willing to agree to anything [30].

#### The thematic pattern of barriers and facilitators

It is notable that the identified barriers and facilitators include cognitive, emotional, social, practical and instrumental factors.

We identified a smaller number of facilitators than barriers, and three facilitating factors were dominant: the potential for personal benefit; altruism; and trust. Each of these was identified in a majority of the 26 included systematic reviews. These three factors were evidenced across different health settings and different research designs: they appear to be generic factors in being potentially important influences on individuals’ decisions about research participation whatever the context.

Barriers to participation were larger in number and more disparate. Their influence also appears to relate to the research design and to individual circumstances. For example, patients had stated treatment preferences or a current stable state of health, both of which might be disrupted by research involving a change to treatment. In patients with HIV or mental illness, research participation could be seen as threatening to self-identity or other’s perception of them. Distrust of research was reported and was often culturally specific, being reported most often in minority and ‘low power’ population groups. Practical difficulties associated with research were related to individuals’ circumstances, such as the impact of research on transport costs, childcare or paid work: the impact of these factors on participation will vary considerably across the population. Many stated barriers were specific to trial-based research, with expressed dislike of randomisation, uncertainty and possible treatment change.

### Determinants and their links to the Theoretical Domains Framework

The identified barriers and facilitators from the 26 systematic reviews each link to at least one TDF domain, although there is a clustering on knowledge, social influences, optimism (or pessimism), goals and beliefs about consequences (see Table 2). Each of these domains was then mapped to the overarching constructs outlined in the COM-B model. Amongst the inductively identified facilitators of research participation, the three most commonly included were personal benefit; altruism; and trust. All the facilitators including the three most common ones map to different facets of the Motivation component of the COM-B model.

Amongst the 11 inductively identified barriers, all are linked to Motivation facets (both reflective and automatic), with two also linked to Opportunities. One barrier was linked to Physical Capabilities. The two factors that could operate either as facilitators or barriers (other people’s influence; information quality and participant knowledge) were mapped to Motivation and Opportunity components, respectively.

### Reported reasons for/against research participation and links to empirical recruitment research

There is a lack of overlap between the barriers and facilitators we identified and the interventions tested, both in terms of the distribution of studied strategies and their impact. Whilst treatment preference was an important barrier to participation, only one study tested a strategy (patient preference trial design) which could be mapped to this theme. For a number of identified barriers, including condition stigma and distrust, we identified no related interventions. Similarly, no identified studies appeared to analyse strategies which may improve recruitment by impacting on altruistic motives. Additionally, there were no tested interventions linked to the patient’s confidence in the physician and the influence of family and/or friends, although the influence of recruitment via the Church or endorsements by previous participants has been studied.

Three tested recruitment strategies (phone reminders, recruitment primer letters, increased contact during recruitment in person or by phone) were not linked to any identified psychosocial determinants. Phone reminders act as a prompt to memory, whilst primer letters act by raising awareness, with neither cited as a barrier to participation. Increased contact during recruitment could potentially act on knowledge, although its intended action is not made clear. A fifth strategy not linked to the identified psychosocial determinants (strategies aimed at recruiters or recruitment sites) is intended to change the behaviour of recruiters, not participants. Also of note is that our overview identified three systematic reviews that investigated barriers or facilitators in relation to recruitment to paediatric research, and yet only one of the intervention studies included in the Treweek review [8] assessed recruitment to paediatric trials.

## Discussion

### Statement of principal findings

This overview found that a small number of psychosocial facilitators were evident, spanning settings and demographic groups. Psychosocial barriers were larger in number and more sensitive to research context and individual circumstances. When psychosocial determinants were mapped to the TDF and COM-B model, there was clustering on the opportunity and motivation domains. When determinants were mapped to recruitment strategies, there was incomplete overlap, and a number of determinants had no clear link to any evaluated recruitment interventions.

### Strengths and weaknesses of the study

Overviews offer the potential for clarity in areas of significant systematic review activity [44] and may also identify consistencies and inconsistencies in primary evidence, and relative levels of importance [45]. This overview has clarified the psychosocial determinants of research participation and also identified clear opportunities to develop recruitment science by drawing on theory and empirical evidence. Some strengths of this overview are the searching of multiple databases, the use of dual independent assessors throughout and the inclusion of reviews not published in English. Excluding low-quality reviews (*n* = 4) increased rigour but reduced the number of included reviews and primary studies. We also excluded four reviews when we could not separate findings derived from patients and practitioners, and 22 reviews because less than two thirds of their included studies reported real research scenarios; in both cases this potentially reduced the total evidence base. In none of these cases do we think the exclusions have introduced bias or significantly limited findings. For example, AMSTAR has recently been shown to identify low-quality reviews for exclusion from an overview, without introducing bias [46]. Although overviews have been published for more than a decade, they continue to be subject to methodological debate, particularly around primary study duplication [43, 47]. Fifty-six (11.6%) of the primary studies in this overview were included in more than one review, and we did not adjust the findings to take account of this; our rationale was that we were reporting findings thematically and not undertaking pooling of quantitative data. However, primary study duplication may have led to overstatement of some determinants. The overview focused on barriers and facilitators of participation in health research broadly, but it mapped them against recruitment interventions to trials. We acknowledge the mismatch, but there is no recently published systematic review of interventions to increase participation in non-trial health research.

### Strengths and weaknesses in relation to other studies, highlighting important differences in results

For the first time this overview has brought together evidence on the determinants of health research participation from a wide range of settings and methods. One of its key contributions is to clarify the relatively small number of psychosocial factors that have a consistent, positive influence on patients’ decisions. That these three factors (potential for personal benefit, altruism and trust) are key determinants is an important insight, as is the finding that their influence spans health setting and type of research design. They are evident both in qualitative and quantitative primary studies. In itself this speaks to the value of an overview; this pattern would not be evident in an individual systematic review. The barriers identified by the overview are more context-specific, such as the stigma associated with certain conditions, the practical demands that some research can place on participants and also the suspicions felt by some minority ethnic groups about some clinical research. Again, this finding of context-specificity could not be derived from a single-setting systematic review. Mapping identified psychosocial determinants onto a theoretical framework and assessing the overlap of psychosocial determinants with recruitment interventions has provided novel insights.

### Meaning of the study: possible explanations and implications for clinicians and policymakers

Clarification of the main themes in psychosocial determination is itself useful knowledge for a number of stakeholders, including clinicians, researchers and research ethics organisations. It should be possible for researchers to use this knowledge to enhance recruitment, for example by drawing on the power of trust by using personal endorsements or role models from the same cultural background, or by acknowledging the credibility of the people and organisations involved in the research. However, interventions emphasising altruism (the potential for others to benefit from one’s actions) or the potential for personal benefit (when that could be uncertain in research, particularly in a controlled trial) could raise ethical challenges. Furthermore, it was notable that social influences—the effects of family, the doctor and other people seen as important—could act either as a barrier or a facilitator for a person deciding whether to participate in health research.

Identifying barriers to research should make it possible for adjustments to be made to the design and operationalisation of research, particularly if barriers are specific to study design and setting. Participant information, participant knowledge and social influences were found to act both as barriers and facilitators, and this perhaps presents a problem. First, researchers are unlikely to be able to control for social influences; second, information to inform research participation is mostly universal in provision and highly regulated [48], and yet amongst patients there can be strong, individual preferences for the quantity and complexity of information [49–52]. The opposing forces of universalism and individual preferences can be hard to reconcile, although digital provision does increase the potential for information to be tailored or personalised, whether by the originator or recipient.

Mapping identified determinants to theory offers the potential for greater understanding of individuals’ decisions and opportunities for linkage. Carey et al. (2019) systematically mapped the evidence for behaviour change techniques to mechanisms of action from hundreds of research studies [16]. They showed that the mechanism of action ‘beliefs about consequences’, which we linked to a number of participation determinants, had strong empirical links to the following: information about health consequences; information about social and environmental consequences; pros and cons; information about emotional consequences; and comparative imagining for future outcomes. Any of these could guide recruitment interventions.

The lack of complete overlap between psychosocial determinants and empirical recruitment research also offers the potential to guide intervention development. Recruitment interventions could focus on altruism (noting potential ethical concerns); the stigma associated with the health condition; or distrust of research or researchers—none of which has been tested in recruitment interventions. Furthermore many other determinants, such as treatment preferences; fear and perceived risk; confidence or trust in the physician or research; and desire for choice, have had little or no evaluation in recruitment research.

### Unanswered questions and future research

Mapping of the psychosocial determinants onto recruitment interventions offers the potential for new research, as outlined above, and the possibility of applying an empirical framework to explain and predict the actions of recruitment interventions. Whilst the application of the TDF and COM-B models to the identified psychosocial determinants has produced new insights, it assumes that research participation is an explainable behaviour, and this assumption would benefit from empirical and theoretical evaluation. Very many published recruitment interventions have been atheoretical and lack clarity about possible mechanisms of action; thus, there is an opportunity in future recruitment research to incorporate the growing science of behaviour change.

This overview included 26 systematic reviews reporting more than 400 primary studies, but areas for development remain. For example, almost two thirds (59.9%) of primary studies in the reviews had been undertaken in just four countries, all of them English-speaking (although a proportion of reviews did not report country of origin). It was surprising that separate reviews had not been conducted in primary care settings or healthy person screening, since the experience of research participation may be very different from emergency care or long-term health conditions, for example. We excluded much research using hypothetical scenarios and, given the volume of real scenario research that we did include, the value of new hypothetical scenario research is questionable. Finally a significant proportion of the included reviews and primary studies used qualitative methods, and it is possible that narrative synthesis, an area of rapid methodological development, has potential to offer new insights into the determinants of research participation.

## Conclusions

We identified a number of psychosocial barriers and facilitators to research participation, of which several spanned patient groups and settings, whilst the effect of others was more context-specific. These could be addressed by researchers when planning and implementing recruitment to studies. There is a need for more research to identify effective recruitment strategies that draw on theory and the psychosocial facilitators and barriers identified in this overview.

## Supplementary information

**Additional file 1.** MEDLINE search strategy.

## Data Availability

Data are available from the corresponding author on reasonable request.
