# Correction to: Why do patients take part in research? An overview of systematic reviews of psychosocial barriers and facilitators

**DOI:** 10.1186/s13063-020-04793-2

**Published:** 2020-10-08

**Authors:** Rebecca Sheridan, Jacqueline Martin-Kerry, Joanna Hudson, Adwoa Parker, Peter Bower, Peter Knapp

**Affiliations:** 1 University of York, York, UK; 2 Kings College London, London, UK; 3 University of Manchester, Manchester, UK; 4 University of York and the Hull York Medical School, York, UK

**Correction to: Trials 21, 259 (2020)**

**https://doi.org/10.1186/s13063-020-4197-3**

Following publication of the original article [1], we were notified that a few lines in Table 2 were misaligned. The correct Table 2 is presented below.

**Table 2:** Identified psychosocial facilitators and barriers to research participation, mapped to tested recruitment interventions and the Theoretical Domains Framework. [Key: (−) Negative effect on recruitment; (?) Uncertain effect on recruitment]

Identified theme	Systematic Reviews reporting the theme	Domain (components) of the TDF. (from Cane et al, 2012)	Interventions which probably affect recruitment to research. (from Treweek et al, 2018)	Interventions shown not to affect recruitment to research, or with uncertain effects. (from Treweek et al, 2018)
***Facilitators***				
Personal benefit (including therapeutic benefits; closer monitoring; access to new treatments; gaining knowledge of own health)	Dhalla, 2014; Fayter, 2007; Fisher, 2011; Grand, 2012; Hughes-Morley, 2015; Liljas, 2017; Limkakeng, 2013a; Limkakeng, 2013b; McCann, 2007; McCann, 2013; Martinsen, 2016; Nalubega, 2015; Nievaard, 2004; Nobile, 2013; Quay, 2017; Tromp, 2016.	Optimism (Reflective Motivation) Beliefs about consequences (Reflective Motivation) Goals (Reflective Motivation) Reinforcement (Automatic Motivation)	Mentioning scarcity of trial places. Positive framing of potential treatment benefits.	Patient preference trial design.
Altruism (including benefits to science; helping others)	Dhalla, 2014; Fayter 2007; Fisher 2011; Hughes-Morley 2015; Limkakeng, 2013a; Limkakeng, 2013b; Martinsen, 2016; McCann, 2007; McCann, 2013; Nalubega, 2015; Nobile, 2013; Nievaard, 2004; Quay, 2017; Tromp, 2016.	Belief about consequences (Reflective Motivation) Social influences (Social Opportunity)		
Financial benefit or incentives	Limkakeng, 2013a; Nalubega, 2015; Tromp, 2016.	Reinforcement (Automatic Motivation)	Financial incentives	
Participant’s knowledge of the research	Fayter 2007; Crane, 2017; Glover, 2015.	Knowledge (Psychological Capability)	Enclosing questionnaire on study method.	Researcher reading out information (?). Easy to read consent form. Optimising information through user testing or user feedback. Brief patient information leaflet. Providing information by phone. Providing information by video (?). Providing audio record of recruitment discussion (?). Providing booklet on trial methods (?). Total or discretionary information disclosure (?). Educational package on study.
Confidence or trust in the physician or the research	Crane, 2017; Grand, 2012; Hughes-Morley, 2015; Liljas, 2017; Limkakeng, 2013a; Limkakeng, 2013b; Martinsen, 2016; McCann, 2007; McCann, 2013; Nievaard, 2004; Nobile, 2013.	Social or Professional Role & Identity (Reflective or Automatic Motivation)	Endorsements of previous participants.	
Influence of family or friends	Hughes-Morley, 2015; Gad 2018; Liljas, 2017; Limkakeng, 2013a; Tromp, 2016.	Social influences (Social Opportunity)	Endorsements of previous participants. Recruitment at a church.	Recruiters from same ethnic group as participants (?).
Low burden or convenient research	Limkakeng, 2013a; Nobile, 2013; Tromp, 2016.	Belief about consequences (Reflective Motivation). Environmental context and resources (Physical Opportunity)		Two stage randomisation method.
***Barriers***				
Treatment preferences (for specific therapy; against placebo)	Fayter, 2007; Grand, 2012; McCann, 2007; Prescott, 1999; Tromp, 2016.	Goals (Reflective Motivation).	Open trial design.	Patient preference trial design.
Stigma associated with health condition	Dhalla, 2013; Hughes-Morley, 2015; Nalubega, 2015; Woodall, 2010; Quay, 2017.	Emotion (Automatic Motivation). Belief about consequences (Reflective Motivation).		
Distrust of research or researchers (particularly among ethnic minorities)	Glover, 2015; Hughes-Morley, 2015; Limkakeng, 2013a; Limkakeng, 2013b; McCann, 2007; Nalubega, 2015; Quay, 2017; Tromp, 2016; Woodall, 2010.	Belief about consequences (Reflective Motivation). Optimism / pessimism (Reflective Motivation).		
Lack of knowledge of the research	Glover, 2015; Limkakeng, 2013a; Limkakeng, 2013b; Prescott, 1999; Tromp, 2016.		Enclosing questionnaire on study method.	Researcher reading out information (?). Easy to read consent form. Optimising information through user testing or user feedback. Brief patient information leaflet. Providing information by phone. Providing information by video (?). Providing audio record of recruitment discussion (?). Providing booklet on trial methods (?). Total or discretionary information disclosure (?). Educational package on study.
Fear and perceived risk (to health, of experimental treatment or adverse effects; to personal consequences)	Dhalla 2013; Fisher 2011; Grand, 2012; Hughes-Morley, 2015; Martinsen, 2016; McCann, 2013; Nalubega, 2015; Nievaard, 2004; Quay, 2017; Tromp, 2016; Woodall, 2010.	Emotion (Automatic Motivation). Belief about consequences (Reflective Motivation).	Emphasising pain in information (−).	Emphasising risk in information.
Aversion to randomisation	Hughes-Morley, 2015; McCann, 2007; McCann, 2013; Nievaard, 2004; Tromp, 2016.	Belief about consequences (Reflective Motivation).		Cluster trial design.
Practical difficulties (including additional procedures or appointments; transport; costs; work or caring responsibilities)	Fayter, 2007; Glover, 2015; Grand, 2012; Hughes-Morley, 2015; Liljas, 2017; Martinsen, 2016; McCann, 2007; Prescott, 1999; Quay, 2017; Tromp, 2016; Woodall, 2010.	Reinforcement (Automatic Motivation). Environmental context and resources (Physical Opportunity).	Financial incentives. Internet-based data collection (−).	Two stage randomisation method. Email (not postal) invitations.
Desire for choice	Grand 2012; Fisher 2011; Tromp 2016.	Belief about consequences (Reflective Motivation).		Patient preference trial design.
Uncertainty (particularly in relation to trials; its links to randomisation)	Fayter, 2007; Fisher 2011; Nievaard, 2004; Prescott, 1999.	Belief about consequences (Reflective Motivation).		Patient preference trial design.
Influence of physician or family	Fayter, 2007; Liljas, 2017; Prescott, 1999.	Social or Professional Role & Identity (Reflective or Automatic Motivation) Social influences (Social Opportunity)	Endorsements of previous participants.	
Personal health	Hughes-Morley, 2015; Liljas, 2017; Limkakeng, 2013b; Woodall, 2010.	Environmental context and resources (Physical Opportunity). Belief about consequences (Reflective Motivation).		
			**Interventions not related to any identified barriers or facilitators:** **Interventions which probably affect recruitment to research. (from Treweek et al, 2018).**	**Interventions not related to any identified barriers or facilitators:** **Interventions shown not to affect recruitment to research, or with uncertain effects. (from Treweek et al, 2018).**
			Telephone reminders.	Sending recruitment primer letter.
			Opt-out consent.	Recruitment method involving more contact in person or by phone.
				Interventions aimed at recruiters or recruitment sites (?).
