# Supplementary material for: Why do patients take part in research? An overview of systematic reviews of psychosocial barriers and facilitators
Source: Trials. 2020 Mar 12;21:259. doi: 10.1186/s13063-020-4197-3 (PMC7069042; doi:10.1186/s13063-020-4197-3)
Supplement: Supplementary file 1 — Additional file 1. MEDLINE search strategy. [file 13063_2020_4197_MOESM1_ESM.docx]

**Additional file 1: MEDLINE search strategy.**

Database: Epub Ahead of Print, In-Process & Other Non-Indexed Citations, Ovid MEDLINE(R) Daily and Ovid MEDLINE(R) <1946 to Present>

Search Strategy:

--------------------------------------------------------------------------------

1 ((difficult$ or problem$ or obstacle$ or barrier$) adj2 (join or joins or joining or joined or enter or enters or entered or entry or sign up or signed up or signing up or opt in or opt out or opting in or opting out or opted in or opted out) adj2 (trial$ or study or studies or research or rct or rcts)).ti,ab. (5)

2 ((deter or deters or deterrent or discourag$ or adverse$ or impediment or failure or impede) adj2 (join or joins or joining or joined or enter or enters or entered or entry or sign up or signed up or signing up or opt in or opt out or opting in or opting out or opted in or opted out) adj2 (trial$ or study or studies or research or rct or rcts)).ti,ab. (13)

3 ((attitude$ or knowledge or understand$ or decision$ or process$ or strateg$ or reason$ or factor$ or incentive or benefit$ or personal gain$) adj2 (join or joins or joining or joined or enter or enters or entered or entry or sign up or signed up or signing up or opt in or opt out or opting in or opting out or opted in or opted out) adj2 (trial$ or study or studies or research or rct or rcts)).ti,ab. (61)

4 ((determin$ or perception$ or perceiv$ or perspective$ or preference$ or view$ or belief$ or experienc$) adj2 (join or joins or joining or joined or enter or enters or entered or entry or sign up or signed up or signing up or opt in or opt out or opting in or opting out opted in or opted out) adj2 (trial$ or study or studies or research or rct or rcts)).ti,ab. (34)

5 ((willing$ or ready or able or readiness or agree$ or consent or permission or assent or volunteer$ or voluntar$ or altruis$ or permit$ or choose or choice or chose) adj2 (join or joins or joining or joined or enter or enters or entered or entry or sign up or signed up or signing up or opt in or opt out or opting in or opting out or opted in or opted out) adj2 (trial$ or study or studies or research or rct or rcts)).ti,ab. (180)

6 ((commitment or committed or accept or acceptance or nonacceptance or offer or offers or offering or offered) adj2 (join or joins or joining or joined or enter or enters or entered or entry or sign up or signed up or signing up or opt in or opt out or opting in or opting out or opted in or opted out) adj2 (trial$ or study or studies or research or rct or rcts)).ti,ab. (9)

7 ((facilitat$ or motivat$ or incentiv$ or maximis$ or technique$ or enhanc$ or encourag$) adj2 (join or joins or joining or joined or enter or enters or entered or entry or sign up or signed up or signing up or opt in or opt out or opting in or opting out or opted in or opted out) adj2 (trial$ or study or studies or research or rct or rcts)).ti,ab. (30)

8 ((selection or preselection or improve or improves or improved or improving or increas$ or eligible or eligibility) adj2 (join or joins or joining or joined or enter or enters or entered or entry or sign up or signed up or signing up or opt in or opt out or opting in or opting out opted in or opted out) adj2 (trial$ or study or studies or research or rct or rcts)).ti,ab. (162)

9 ((refus$ or declin$ or coerce or unwilling$ or uncertain$ or consider$ or discourag$ or reluctan$ or decrease$ or decreasing) adj2 (join or joins or joining or joined or enter or enters or entered or entry or sign up or signed up or signing up or opt in or opt out or opting in or opting out or opted in or opted out) adj2 (trial$ or study or studies or research or rct or rcts)).ti,ab. (60)

10 or/1-9 (548)

11 exp clinical trial/ (743565)

12 clinical trial.pt. (502211)

13 exp Clinical Trials as Topic/ (294420)

14 (trial or trials or study or studies or research or rct or rcts).ti. (1850369)

15 Interviews as Topic/ (47681)

16 exp "Surveys and Questionnaires"/ (787301)

17 or/11-16 (3188720)

18 Patient Participation/ (20077)

19 Refusal to Participate/ (556)

20 exp Research Subjects/ (11012)

21 *Informed Consent/ (15182)

22 *Patient Selection/ (14460)

23 ((difficult$ or problem$ or obstacle$ or barrier$) adj2 (accru$ or recruit$ or enrol$ or participat$ or nonparticipat$ or enlist$)).ti,ab. (3626)

24 ((deter or deters or deterrent or discourag$ or adverse$ or impediment or failure or impede) adj2 (accru$ or recruit$ or enrol$ or participat$ or nonparticipat$ or enlist$)).ti,ab. (1055)

25 ((attitude$ or knowledge or understand$ or decision$ or process$ or strateg$ or reason$ or factor$ or incentive or benefit$ or personal gain$) adj2 (accru$ or recruit$ or enrol$ or participat$ or nonparticipat$ or enlist$)).ti,ab. (15073)

26 ((determin$ or perception$ or perceiv$ or perspective$ or preference$ or view$ or belief$ or experienc$) adj2 (accru$ or recruit$ or enrol$ or participat$ or nonparticipant$ or enlist$)).ti,ab. (4959)

27 ((willing$ or ready or able or readiness or agree$ or consent or permission or assent or volunteer$ or voluntar$ or altruis$ or permit$ or choose or choice or chose) adj2 (accru$ or recruit$ or enrol$ or participat$ or nonparticipat$ or enlist$)).ti,ab. (19862)

28 ((commitment or committed or accept or acceptance or nonacceptance or offer or offers or offering or offered) adj2 (accru$ or recruit$ or enrol$ or participat$ or nonparticipat$ or enlist$)).ti,ab. (960)

29 ((facilitat$ or motivat$ or incentiv$ or maximis$ or technique$ or enhanc$ or encourag$) adj2 (accru$ or recruit$ or enrol$ or participat$ or nonparticipat$ or enlist$)).ti,ab. (7876)

30 ((selection or preselection or improve or improves or improved or improving or increas$ or eligible or eligibility) adj2 (accru$ or recruit$ or enrol$ or participat$ or nonparticipat$ or enlist$)).ti,ab. (13275)

31 ((refus$ or declin$ or coerce or unwilling$ or uncertain$ or consider$ or discourag$ or reluctan$ or decrease$ or decreasing) adj2 (accru$ or recruit$ or enrol$ or participat$ or nonparticipat$ or enlist$)).ti,ab. (5046)

32 or/18-31 (118177)

33 17 and 32 (46138)

34 10 or 33 (46607)

35 systematic$ review$.ti,ab. (88181)

36 meta-analysis as topic/ (15027)

37 meta-analytic$.ti,ab. (4847)

38 meta-analysis.ti,ab,pt. (101687)

39 metanalysis.ti,ab. (147)

40 metaanalysis.ti,ab. (1309)

41 meta analysis.ti,ab. (81570)

42 meta-synthesis.ti,ab. (397)

43 metasynthesis.ti,ab. (191)

44 meta synthesis.ti,ab. (397)

45 meta-regression.ti,ab. (3890)

46 metaregression.ti,ab. (396)

47 meta regression.ti,ab. (3890)

48 (synthes$ adj3 literature).ti,ab. (1932)

49 (synthes$ adj3 evidence).ti,ab. (5601)

50 integrative review.ti,ab. (1400)

51 data synthesis.ti,ab. (8497)

52 (research synthesis or narrative synthesis).ti,ab. (1279)

53 (systematic study or systematic studies).ti,ab. (9182)

54 (systematic comparison$ or systematic overview$).ti,ab. (2404)

55 evidence based review.ti,ab. (1578)

56 comprehensive review.ti,ab. (9292)

57 critical review.ti,ab. (12686)

58 quantitative review.ti,ab. (552)

59 structured review.ti,ab. (593)

60 realist review.ti,ab. (124)

61 realist synthesis.ti,ab. (90)

62 or/35-61 (211291)

63 review.pt. (2141613)

64 medline.ab. (75700)

65 pubmed.ab. (54343)

66 cochrane.ab. (45204)

67 embase.ab. (46353)

68 cinahl.ab. (14888)

69 psyc?lit.ab. (896)

70 psyc?info.ab. (13300)

71 (literature adj3 search$).ab. (36410)

72 (database$ adj3 search$).ab. (34579)

73 (bibliographic adj3 search$).ab. (1630)

74 (electronic adj3 search$).ab. (12834)

75 (electronic adj3 database$).ab. (15987)

76 (computeri?ed adj3 search$).ab. (3012)

77 (internet adj3 search$).ab. (2246)

78 included studies.ab. (11387)

79 (inclusion adj3 studies).ab. (9421)

80 inclusion criteria.ab. (50547)

81 selection criteria.ab. (23792)

82 predefined criteria.ab. (1388)

83 predetermined criteria.ab. (834)

84 (assess$ adj3 (quality or validity)).ab. (52923)

85 (select$ adj3 (study or studies)).ab. (47257)

86 (data adj3 extract$).ab. (38655)

87 extracted data.ab. (9056)

88 (data adj2 abstracted).ab. (3934)

89 (data adj3 abstraction).ab. (1117)

90 published intervention$.ab. (135)

91 ((study or studies) adj2 evaluat$).ab. (132356)

92 (intervention$ adj2 evaluat$).ab. (7778)

93 confidence interval$.ab. (287118)

94 heterogeneity.ab. (115815)

95 pooled.ab. (58967)

96 pooling.ab. (9137)

97 odds ratio$.ab. (190095)

98 (Jadad or coding).ab. (142280)

99 or/64-98 (1012508)

100 63 and 99 (157343)

101 review.ti. (325158)

102 101 and 99 (72800)

103 (review$ adj4 (papers or trials or studies or evidence or intervention$ or evaluation$)).ti,ab. (130563)

104 62 or 100 or 102 or 103 (376121)

105 letter.pt. (927443)

106 editorial.pt. (408795)

107 comment.pt. (672505)

108 105 or 106 or 107 (1503322)

109 104 not 108 (366724)

110 exp animals/ not humans/ (4260951)

111 109 not 110 (356148)

112 34 and 111 (1507)
